# Conventional Versus Accelerated Dry‐Aged Meat: Chemical and Sensory Profiles of *Longissimus lumborum* Muscles From Nellore and Angus × Nellore Crossbreeds

**DOI:** 10.1111/1750-3841.70577

**Published:** 2025-09-24

**Authors:** Angélica Sousa Guimarães, Jéssica Sousa Guimarães, Lorrany Ramos do Carmo, Bruna Fernandes Andrade, Vanelle Maria da Silva, Alcinéia de Lemos Souza Ramos, Eduardo Mendes Ramos

**Affiliations:** ^1^ Departamento de Ciência dos Alimentos, Escola de Ciências Agrárias de Lavras Universidade Federal de Lavras Lavras Minas Gerais Brasil; ^2^ Instituto de Ciências Exatas e Tecnológicas Universidade Federal de Viçosa Florestal Minas Gerais Brasil

**Keywords:** amino acids, descriptive sensory, fatty acids, freezing/thawing, multiple factors analysis, volatile compounds

## Abstract

The objective of this study was to compare the effects of dry aging by conventional (28 days aged; NF28d) and accelerated (frozen/thawed and 14 days aged; FT14d) processes on the sensory and chemical (fatty acids, FA; volatile organic compounds, VOC; and amino acids; AA) profiles of striploins from Nellore and crossbreed F1 Angus × Nellore (F1Angus) bulls. The FT14d Nellore FA profile was close to the profile of the F1Angus samples in both systems (FT14d and NF28d), but F1Angus meat had a higher polyunsaturated/saturated ratio than Nellore's meat (0.42 vs. 0.31).

FT14d changed the VOC profile of grilled meat and exudate, being the exudate VOC most related to the sensory profile. Nellore FT14d was more related to AA glycine, alanine, and glutamic acid, and F1Angus (FT14d and NF28) with leucine and isoleucine but all three samples were related to commonly dry‐aged beef VOC acetoin, 2‐methylbutanal, and 3‐methylbutanal. Although each aging process generated distinct sensory profiles, the genotype was the determining factor in discriminating against beef samples, particularly for the texture attributes. While acceptance scores were unaffected by the aging processes within each genotype, higher overall acceptance scores were observed for F1Angus and Nellore FT14d samples. In conclusion, the accelerated aging treatment provided F1Angus beef samples with a chemical and sensory profile like those obtained by the conventional process. Moreover, an improvement in the sensory quality of lower‐marbling beef (Nellore) was observed with accelerated aging, characterizing a viable and competitive technology alternative for commercializing the meat from these animals.

**Practical Applications:**

The data from this study demonstrate that accelerated dry‐aging improves the flavor and texture of leaner meats, such as Nellore beef, in less time. This approach offers a cost‐effective alternative for the meat industry while delivering a more flavorful and tender product to consumers.

## 1 Introduction

The dry‐aged beef has attracted the interest of retailers, the food industry, and restaurants as a market trend since it's a premium product of high‐added value appreciated by exclusive market niches (Haddad et al. 2022). The dry aging process involves storing unpacked primal or sub‐primal cuts at controlled temperature, airflow, and relative humidity for up to 60 days to improve or intensify the meat flavor generating a characteristic meaty descriptive sensory profile, having robust, roast, umami, buttery, sweet, nutty, and earthy taste (Campbell et al. 2001; O'Quinn et al. 2016). Aging periods of 25–35 days are sufficient to obtain the desired effects of dry aging, such as an ideal balance of tenderness, flavor, and juiciness (Savell 2008; Dashdorj et al. 2016) and have been suggested by the U.S. Meat Export Federation (USMEF 2020) marketing dry‐aged meat internationally.

To generate meat of high added value, the dry aging process is preferably performed with high‐marbled meat, such as from European beef cattle breeds (*Bos taurus*), to generate flavorful and juicy meats (Dashdorj et al. 2016; Iida et al. 2016). However, the dry aging process has also been suggested as an efficient alternative to enhance the sensory quality of low‐marbled meat, such as zebu (*Bos indicus*) cattle (Smith et al. 2008; Berger et al. 2018; Guimarães et al. 2024). It is estimated that 80% of Brazil's cattle herd directed to meat production is made of zebu cattle, particularly Nellore breeds, which is based on their productivity, natural disease resistance, and heat tolerance (Centro de Referência da Pecuária Brasileira 2023). Despite those market characteristics and the fact that the Brazilian production and commercializing of dry‐aged meat is still incipient, recent research has shown that 62% of meat producers are concerned about beef cattle genotypes for dry aging processing. Most of these producers (96%) preferred beef cuts of European breeds or their crossbreeds with zebu cattle (Rezende‐de‐Souza et al. 2021).

Besides the lower marbling fat levels, zebu cattle's meat quality is recognized as lower than that of European breeds because of the reduced proteolysis during aging, which demands longer aging processes to achieve the desired tender texture (Aroeira et al. 2016). However, recent publications have proposed freezing and thawing zebu meat before dry aging as an alternative to accelerate the aging process (Haddad et al. 2022; Guimarães et al. 2024). Those publications have demonstrated that the characteristic aromatic profile is maintained, and instrumental and sensory tenderness obtained after 14 days of dry aging is comparable to that obtained at 28 days of conventional (nonfrozen) aging. Those improvements were achieved without compromising microbiological safety or increasing mass losses, as the losses at 14 days of accelerated aging were comparable to those observed after 28 days of conventional. Even though the cited publications reported they achieved an effective tenderness in a shorter aging time, the effects of the accelerated process on the descriptive sensory profile and consumer acceptance were not evaluated.

It is also important to highlight that several factors influence the formation of compounds responsible for meat flavor and aroma, such as genetic group and aging method (Rezende‐de‐Souza et al. 2021). Barcellos et al. (2017) reported that meat from Nellore cattle has a higher proportion of saturated fatty acids (FA), lower monounsaturated FAs, and a similar proportion of polyunsaturated FAs compared to Angus × Nellore crossbreeds. In addition, amino acids (AA) such as tryptophan, phenylalanine, valine, tyrosine, glutamate, isoleucine (ILE), and leucine (LEU) are significantly more abundant in dry‐aged beef (Y. H. B. Kim et al. 2016). However, the aging time plays a key role in forming these and other flavor‐related compounds. Recent studies have shown that it is possible to achieve a similar profile of volatile and aromatic compounds through accelerated dry aging, with the generation of characteristic components such as 2‐methylbutanal and 3‐methylbutanal (Haddad et al. 2022; Guimarães et al. 2024).

In this context, the accelerated dry aging process may be an alternative to reduce the tenderness time and to meet consumers’ expectations for meat quality traits, specifically for meat from zebu cattle. Nevertheless, publications describing sensory attributes and aromatic components of meat from zebu cattle are scarce, particularly those that evaluated previously frozen/thawed meat. Thus, this study aimed to evaluate the effects of the accelerated dry aging process on the descriptive sensory profile and consumer acceptance of meat from Nellore and crossbreed F1 Angus × Nellore bulls. The evaluated beef samples comprise meat of distinct marbling levels consumed in different markets in Brazil and foreign countries. Moreover, this study described the relationship between these effects with the quality parameters, such as FA, volatile organic compounds (VOC), and AA profiles.

## 2 Materials and Methods

### 2.1 Samples Characterization

Bone‐in striploins samples (cut from the 12th and 13th rib to first lumbar vertebra) from carcasses with high covering fat levels (> 10 mm) of three Nellore breed bulls (24 months old and 305 kg of average carcass weight) and three crossbreed F1 Angus × Nellore bulls (F1Angus; 30 months old and 372 kg of average carcass weight) were collected within 48 h postmortem from a commercial slaughterhouse located in Campo Belo city, MG, approximately 60 km from the Lavras, MG. The cuts were from commercial animals not specifically intended for this study. They were raised under pasture conditions switching to commercial indoor conditions (90 days) before slaughtering. The fat cover thickness and the loin (M. *Longissimus lumborum*; LL) eye area from the cut between 12th and 13th rib were measured as recommended by the USDA (2017). The whole bone‐in striploins were packed in plastic films (not vacuum‐sealed) and transferred into thermic boxes containing ice cubes to the Laboratory of Meat Science (LabCarnes) at UFLA. The transport was completed in approximately 1 h, ensuring proper temperature control and maintaining sample integrity.

### 2.2 Aging Conditions

The pH of each striploin was measured by direct insertion, at four points along the cut, with a portable pH meter (model 206‐pH2; Testo, Campinas, SP, Brazil) and a sample of the LL muscle in the cranial end was removed for analysis of fat content (marbling) by near infrared (NIR; AOAC 2007‐04 method), in a FoodScamTM device (FOSS Analytical A/S, Hillerod, Denmark). Then, each striploin (average 5.53 pH and 4.23% fat in Nellore lean meat and 5.51 pH and 9.86% fat in F1Angus) was cut into two halves using a band saw, weighed and randomly distributed between the treatments (not frozen, NF; frozen/thawed, FT). The sections used in the FT treatment were individually packed in plastic films (not vacuum‐sealed), frozen in commercial freezers (−18°C) for 14 days and thawed (4°C/24 h) for subsequent aging. The NF‐treated samples were directly submitted to the aging process.

The dry aging process was performed using a climatic chamber (Model EL 131/4; Eletrolab Indústria e Comércio, São Paulo, SP, Brazil) with controlled temperature (4.29 ± 0.67°C) and relative humidity (79.81 ± 9.35%) and without forced airflow (<0.2 m/s). Temperature and relative humidity at the chamber were registered during the process with a digital thermohydrometer (Data Logger HT500; Instrutherm Ltda, São Paulo, SP, Brazil). The NF striploins were aged for 28 days (conventional aging; NF28d) and the FT striploins aged for 14 days (accelerated aging; FT14d), according to Haddad et al. (2022). During aging, the samples’ disposition in the chamber's shelves was alternated every 7 days to avoid a position effect in the treatment.

The dry‐aged striploins were weighed to determine the aging losses (evaporation loss in NF and thawing and evaporation losses in FT samples), and the external crust was trimmed and weighed to obtain the trimming loss. Then, they were cut with a band saw obtaining 15 steaks of approximately 2.50‐cm thickness, like bone‐in individual selling cuts, which were identified, individually vacuum packed (BS420; R.Baião Indústria e Comércio, Ubá, MG, Brazil) in nylon‐polyethylene packaging (90 µm thick; 30–60 cm^3^ m^2^ day^−1^ atm^−1^ oxygen transmission rate), and frozen (−18°C) for subsequent sensory analysis, which occurred 30 days after aging.

### 2.3 Analyses

On the day of the sensory analysis, the steaks were previously thawed (4°C/24 h) and deboned (maintaining the covering fat). The LL muscle from two steaks was ground and pooled to determine the FA profile (*n* = 3 by animal), while the remaining steaks were powdered with 1.5% refined salt (sodium chloride), covered with foil, and cooked in an electric grill pre‐heated to 200°C (Model SCGE; Croydon; Duque de Caxias, RJ, Brazil) until they reached an intern temperature of 70°C. The steak's temperature was monitored by a digital thermometer (Smart Wireless BBQ Thermometer; Chugod, Madrid, Spain) inserted in the geometric center point of each steak. After cooking, the samples exudates from two steaks of each animal were collected, pooled and used for VOC and AA profiles analysis (*n* = 3 by animal).

#### 2.3.1 Fatty Acids Profile

The FA composition analysis was performed in a gas chromatographer after the lipid extraction with a chloroform/methanol (2:1 v/v) solution, as described by Folch et al. (1957), and the esterification (FAME) with 0.5 N potassium chloride solution (in methanol) and esterification reagent (0.5 g ammonium chloride, 0.7 mL sulfuric acid and 14 mL methanol), according to Hartman and Lago (1973). An aliquot (1 µL) of FAME extract in hexane was injected in a Shimadzu chromatographer (GC‐MS QP2010 Plus; Shimadzu, Japan), where the injector temperature was adjusted to 260°C, in split mode 1:20, with linear speed of 20 cm/s, heating ramp from the initial temperature of 140°C (5 min) and a heating rate of 4°C/min until 240°C (30 min). An SP 2560 column (100 m × 0.25 mm intern diameter × 0.20 µm of film thick; Supelco Inc., Bellefonte, PA, USA) was used. The identification of FA was obtained by comparing retention times of chromatograph standards Supelco TM37 FAME Mix (Supelco Inc., Bellefonte, PA, USA). The data was expressed as a percentage of the normalized area of FA and grouped as the total of saturated (SFA), monounsaturated (MUFA), polyunsaturated (PUFA), unsaturated (UFA), and omega‐6 (n6) FAs and their relations.

#### 2.3.2 Volatile Organic Compounds Profile

The VOC profile analysis was performed in the grilled samples and their exudate (obtained immediately after cooking the steaks). The separation and identification of VOC were conducted in a gas chromatographer coupled to a mass spectrometer (GC‐MS QP2010 Plus; Shimadzu, Japan), equipped with an automatic injector for liquids and gases (AOC‐5000; S Shimadzu, Japan) and SLB column (5% phenyl‐ 95% dimethylsiloxane; 30 m × 0.25 mm × 0.25 µm; Supelco Inc., Bellefonte, PA, USA).

The samples (5 g of crushed grilled meat or 4 g of exudate) were weighed and placed in 22 mL vials, sealed with a PTFE silicone septa. The VOC were extracted in the headspace by solid phase microextraction (SPME) using a DVB/CAR/PDMS fiber (divinylbenzene, carboxen, and polydimethylsiloxane; 1 cm, 50–30 µm film thick; Supelco) (Haddad et al. 2022). The samples were pre‐heated for 5 min at 65°C in a heating block, and the fiber was exposed for 20 min in the vial for compound extraction (Gardner and Legako 2018). All subsequent procedures for VOC chromatographic analysis and compound identification followed the method described by Haddad et al. (2022).

#### 2.3.3 Amino Acids Profile

The AA profile was determined by high‐performance liquid chromatography (HPLC) in a Shimadzu chromatographer (Shimadzu Corporation, Tokyo, Japan) from the exudate of grilled samples (2.3.8). The samples were hydrolyzed with phenol 3% in HCl 6 M for 24 h at 110°C and subsequently derivatized in a pre‐column with phenyl isothiocyanate. The AA were separated in Agilent's Luna C‐18 reversed‐phase column (100 Å, 5 µM, 250 mm × 4,6 mm; Phenomenex, Torrance, USA) at 50°C and quantified with a UV detector at 254 nm (Hagen et al. 2020). The tryptophan content was determined after alkaline hydrolysis (barium hydroxide 4 M), according to the methodology proposed by Lucas and Sotelo (1980). The content of each AA was expressed as mg/g of protein, determined by the Kjeldahl method (AOAC 928.8) (AOAC 2012).

#### 2.3.4 Sensory Profile and Acceptance

The consumer's perception of sensory and quality features of the dry‐aged beef was evaluated using Check‐All‐That‐Apply (CATA) descriptive test and acceptance test. The sensory analysis was approved by the Human Research Ethics Committee of the Federal University of Lavras (CAAE: 36202520.0.0000.5148), and all panel participants signed a written informed consent form (TCLE).

According to a literature methodology with minor adaptations, CATA and acceptance tests were performed in set (Guimarães et al. 2022). The definition of the attributes present in the CATA test was carried out by a focus group of previously selected evaluators. These evaluators were first recruited based on a basic flavor and aroma recognition test (ISO 8586:2012) and then by a triangular test (ISO 4120:2021). In the triangular tests, two treatments of Nellore beef were used: frozen treatment (−18°C/15 days), thawed (4°C/24 h) and aged (at 4°C) for 15 days in a dry aging and in a wet aging (vacuum‐packed) process. Each evaluator performed a series of at least four triangular tests, which were evaluated through Wald sequential analysis (Meilgaard et al. 1999), to verify the sensory aptitude of candidates with good accuracy in their responses and, thus, selected them to compose the focus group. The candidates’ fitness was verified using a *P* of 0.30 (maximum acceptable disability), *P_1_* of 0.70 (minimum ability accepted), and 0.10 for *α* (probability of accepting a candidate with no sensory accuracy) and β (probability of rejecting a candidate with good sensory accuracy) risks.

After the selection, 17 panelists gathered the characteristics of the CATA form in the focus group using steaks from the four treatments: Nellore and F1Angus conventionally (NF28d) and accelerated dry‐aged (FT14d) beef samples. The panelists identified 26 characteristics grouped in appearance, flavor, and texture (Table 1).

**TABLE 1:** Check‐All‐That‐Apply (CATA) form features list used to characterize the different treatments.

Appearance	Aroma	Taste	Texture
Uniform	Grilled/barbecue	Grilled/barbecue	Firm
Grilled/barbecue	Sweet/caramel	Sweet/caramel	Soft
Brownish	Fatty/buttery	Fatty/buttery	Juicy
Pale	Smoked	Smoked	Dry
Moist	Nutty	Acid	Fibrous
Pinkish at the center	Woody	Metallic	
Apparent fibers	Bloody	Bloody	

The sensory analyses were conducted with 104 untrained consumers with age from 18 to 45, randomly recruited. Among the evaluators, 10% reported they consumed grilled beef once or twice a month/rarely, 60% once or twice a week, and 30% consumed daily. The analyses were performed in one session, conducted in individual cabinets with adequate illumination of white light. All samples evaluated sensorially were salted (1.5% salt) and grilled, covered with foil, as described in Section 2.3. The addition of salt was based on preliminary tests aimed at balancing the enhancement of flavor without masking the characteristic sensory profile of dry‐aged beef. This level was chosen to preserve the robust aroma and taste developed during aging, while also reflecting typical consumer practices, as beef is commonly seasoned with salt before consumption. A transversal cut of 1.0 cm‐thickness was obtained from the grilled sample, portioned in disposable cups, kept in thermic boxes, and served at ∼ 40°C. The panelist judged the four codified meat samples in a balanced order and monadic format. The evaluators were provided with water to clean the taste between evaluations.

Before the evaluation, the panelists were oriented to read the CATA list attributes and indicate appropriate ones to describe each sample. The panelist knew they could select as many attributes from the list as they found applied to evaluated samples. Finally, they were asked to indicate how much they liked or disliked a sample (acceptance test) regarding the attributes of appearance, aroma, taste, texture, and overall acceptance (OA) using a structured hedonic scale of 9 points (from “1 – dislike extremely” to “9 – like extremely”).

### 2.4 Statistical Analysis

For the analysis of FA, AAs profile, and VOCs, the experiment was conducted in a randomized block design (DBC), in which each animal constituted a block (*n* = 3). The main effects were determined using analysis of variance (ANOVA) and post‐hoc comparisons of means were conducted using Tukey test at the 95% confidence level (*p* < 0.05). The relationship of chemical characteristics within the evaluated effects (Nellore and F1Angus in the aging systems NF28d and FT14d) were determined by the principal components analysis (PCA). These analyzes were performed using Statistica 8.0 software (StatSoft Inc., Tulsa, USA).

For the CATA test data, the frequency of citation of each attribute was determined in the samples by counting the times specific terms were used by the consumers (contingency table). From the contingency table, the differences for each attribute among the samples were verified by the Cochran *Q* test (Meyners et al. 2013). The attributes that presented less than 10% of consumers’ citations were excluded (Ares et al. 2010), namely, “nutty” and “bloody” aromas and “acid”, “metallic”, and “bloody” tastes. The correspondence analysis (CA) was calculated over the contingency table to obtain the sensory map of treatments concerning their characteristics (Guimarães et al. 2022). The variance analysis (ANOVA) evaluated the acceptance test data, considering the consumers as a block and the confidence level of 5%. The average of the acceptance tests was compared with the Tukey test.

To comprehend the relationship between the chemical (AA and VOC) and sensory (CATA and OA) datasets, the multifactorial analysis (MFA) was applied to the four treatments of bovine meat. In the presence of qualitative and quantitative variable tables, the global MFA was conducted using the PCA of groups of AA and VOC, the CA for the attributes group from CATA, and the averages of OA. Therefore, the MFA was performed in a table composed of four lines, corresponding to four treatments and four groups of columns, corresponding to 20 AA, 17 VOC (from the exudate), 21 CATA attributes, and the OA averages. The RV‐coefficient, which measures the proximity between two datasets, was calculated to establish a relationship between the groups of evaluated variables. The RV coefficient is a multivariate statistical indicator that varies from 0 (not correlated orthogonal configurations) to 1 (perfect concordance) (Rodrigues et al. 2020). Those analyses were conducted using XLSTAT, version 2022 (Addinsoft, Paris, France).

## 3 Results and Discussion

### 3.1 FA Profile

The FA profile (% of the normalized area), the sum, and their relations of dry‐aged striploin samples of Nellore and F1Angus, using accelerated and conventional processes (FT14d and NF28d, respectively), are described in Table S1. Overall, the FAs profile of F1 Angus meat samples did not statistically differ from the FT14d Nellore, but had a higher PUFA/SFA ratio, meeting the minimum proportion (0.42 against 0.31) considered adequate for determining meat quality and nutritional value (Wood et al. 2004). Although the impact of postmortem aging on the FA profile remains controversial, some studies have reported slight variations depending on factors such as breed, fat content, and aging method. For instance, Y. H. Kim et al. (2019) observed an increase in the PUFA/SFA ratio in high‐marbled Hanwoo beef subjected to dry aging.

**FIGURE 1:**
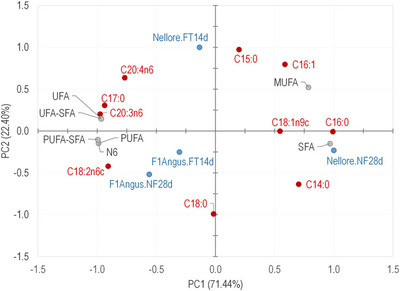
Principal components analysis (PCA) of fatty acid profile of Nellore and crossbreed F1 Angus × Nellore (F1Angus) dry‐aged beef (*L. lumborum*) obtained by accelerated (FT14d) and conventional (NF28d) processes. Fatty acids: C14:0 = myristic; C15:0 = pentadecanoic; C16:0 = palmitic; C16:1 = palmitoleic; C17:0 = heptadecanoic; C18:0 = stearic; C18:1n9c = oleic; C18:2n6c = linoleic; C20:3n6 = eicosatrienoic; C20:4n6 = aracdonic acid. Sum of fatty acids: SFA = saturated; MUFA = monounsaturated; PUFA = polyunsaturated; UFA = unsaturated; and N6 = omega 6.

A PCA graph was also generated for the FA profile to evaluate their effects (Figure 1). PCA's first (PC1) and second (PC2) components represented 93.84% of experimental data variability. PC1 described a data variability higher than 70%. It grouped the samples in NF28d Nellore‐aged samples, characterized by SFA (particularly palmitic and stearic) and MUFA (oleic), and other treatments, characterized by UFA. Although the FT14d process brought the FA profile of Nellore samples closer to crossbreed F1Angus‐aged ones by both systems (FT14d and NF28d), the differences between the genetic groups remain evident. The findings of the present study for the NF28d sample are like those reported by Barcellos et al. (2017), who observed that the meat of wet‐aged Nellore steers had a higher percentage of SFA and a lower percentage of UFA when compared to wet‐aged meat from Angus × Nellore bulls.

### 3.2 Volatile Organic Compounds (VOC) Profile

The VOC profile obtained from beef and exudate samples during the cooking of dry‐aged meat is shown in Table S2. In the grilled meat it was identified 18 compounds classified in the following groups: alcohols (*n* = 4), aldehydes (*n* = 11), ketones (*n* = 2), and pyrazines (*n* = 1). Except for pyrazine and ketones, the VOC sums of each other's classes (alcohols and aldehydes) were always superior in NF28d beef. Concerning the VOC profile of the exudate, although a similar number of compounds were identified (17), the profile differed since acid compounds (*n* = 2) were identified. Besides, compound groups previously identified in the grilled meat were also identified in the exudate, i.e., alcohols (*n* = 2), aldehydes (*n* = 12), ketones (*n* = 1), and pyrazines (*n* = 1). The presence of certain FAs, such as nonanoic and decanoic acids, in the exudate rather than directly in the meat matrix can be explained by their thermal formation and migration during the cooking process. The exudate acts as a carrier of water‐soluble and heat‐released lipids, which can differ in composition from the intact muscle tissue (Khan et al. 2015).

**FIGURE 2:**
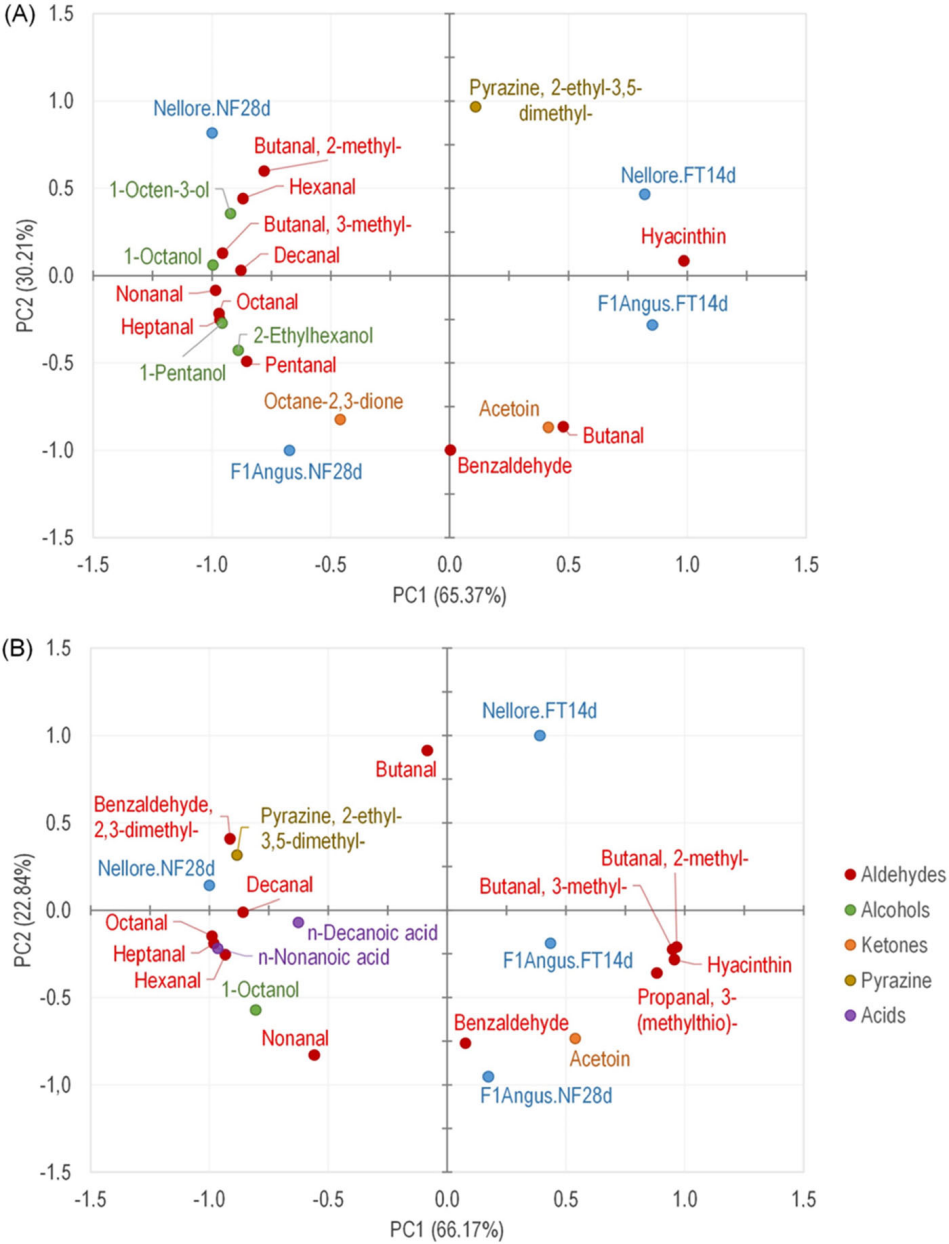
Principal components analysis (PCA) of the volatile organic compounds (VOC) profile of (A) meat and (B) exudate of grilled dry‐aged (*L. lumborum*) from Nellore and crossbreed F1 Angus × Nellore (F1Angus) obtained by accelerated (FT14d) and conventional (NF28d) processes.

From Nellore and F1Angus breeds, the concentration of compounds (× 10^4^ total ions counting), particularly the sum of aldehydes, was superior in the exudate than in the grilled meat, probably because of the aromatic compound's higher concentration and volatilization, including those from meat's lipid fraction. The meat and exudate identified VOC were discriminated against treatments by PCA (Figure 2), where the first (PC1) and second (PC2) components represented 95.58% of experimental data variability for grilled meat and 89.01% for exudate.

Concerning the grilled meat, the accelerated aging modified the VOC profile, and FT14d samples were separated on opposite sides from NF28d samples (PC1), as well as breeds Nellore and F1Angus were separated in PC2 (Figure 2A). Higher number of VOC was related to conventional aging (NF28d), while accelerated aging (FT14d) was characterized mainly by the compounds hyacinthine, butanal, and acetoin (PC1). According to PC2, the volatile compounds butanal and acetoin were more strongly associated with F1 Angus beef, likely due to the higher degree of marbling typical of this breed. Increased intramuscular fat content can enhance the generation of lipid oxidation degradation products during the dry aging process (Kosowska et al. 2017).

A high presence of acetoin ketone in dry‐aged samples was reported by O'Quinn et al. (2016). Da Silva Bernardo et al. (2020) also observed the formation of acetoin ketone in 28‐day dry‐aged Nellore beef samples, either frozen/thawed or not. Haddad et al. (2022), on the other hand, reported a higher correlation of acetoin with frozen/thawed beef aged for 14 days than for unfrozen ones aged for 42 days. The freezing/thawing process can also reduce the stability of meat during storage, as well as contribute to oxidative and microbial rancidity, which also leads to changes in the VOC profile (Guimarães et al. 2024).

A higher number of aldehydes was also related to the conventional aging beef than in the accelerated ones. Specifically, two compounds, 2‐methylbutanal and 3‐methylbutanal, are reported by several authors as characteristic of dry‐aged meat (O'Quinn et al. 2016; Gredell et al. 2018; Da Silva Bernardo et al. 2020; Haddad et al. 2022; Guimarães et al. 2024). These compounds, besides pyrazine‐2‐ethyl‐3,5‐dimethyl and benzaldehyde, are products of the Strecker reaction of ILE, LEU, and phenylalanine (PHE) (Koutsidis et al. 2008). Heptanal, octanal, nonanal, decanal, and octanol are derivatives of oleic acid peroxidation, while butanal and pentanal originate from the linoleic FA (Watanabe et al. 2015; Domínguez et al. 2019; O'Quinn et al. 2016). Therefore, the main compounds formed during aging were products of lipid oxidation, Maillard, Strecker reactions, and their interactions.

Previous studies have reported differences in the VOC profile of dry‐aged Nellore beef depending on whether the samples were frozen/thawed before aging. Haddad et al. (2022) found that acetoin, 2‐methylbutanal, and 3‐methylbutanal were strongly correlated with frozen/thawed beef dry‐aged for 42 days. Da Silva Bernardo et al. (2020) also detected these compounds in 28‐day dry‐aged beef, regardless of prior freezing/thawing. Guimarães et al. (2024) reported that 3‐methylbutanal, octanol, hexanol, and nonanal were more correlated with frozen/thawed samples dry‐aged for 14 days than with 28‐day non‐frozen ones. In our study, only acetoin was related to accelerated aging in grilled beef.

It is worth noting that the exudate VOC profile (Figure 2B) differs from that of grilled beef, being mainly discriminated by PC1 (largest variability; 66.71%) in different groups from those observed for grilled beef samples. FT14d Nellore samples were grouped with F1Angus samples aged with both aging systems (FT14d and NF28d; Figure 2B). Moreover, this group was characterized by the VOC acetoin, 2‐methylbutanal, and 3‐methylbutanal, which was peculiar to dry‐aged grilled meat; especially the last two that were characteristics of the conventional aging.

Regarding the genotype, those were separated into three distinct groups by exudate VOC, with Nellore FT14d samples separated by PC2 (22.84% variability) and FT28d by PC1, both in the opposite quadrants of F1Angus samples. In this case, acetoin and benzaldehyde (related to F1Angus‐aged samples), in contrast with pyrazine‐2‐ethyl‐3,5‐dimethyl (related to Nellore samples), were the VOC that discriminated the genotype samples both the in beef and exudate PCAs.

### 3.3 AA Profile

**FIGURE 3:**
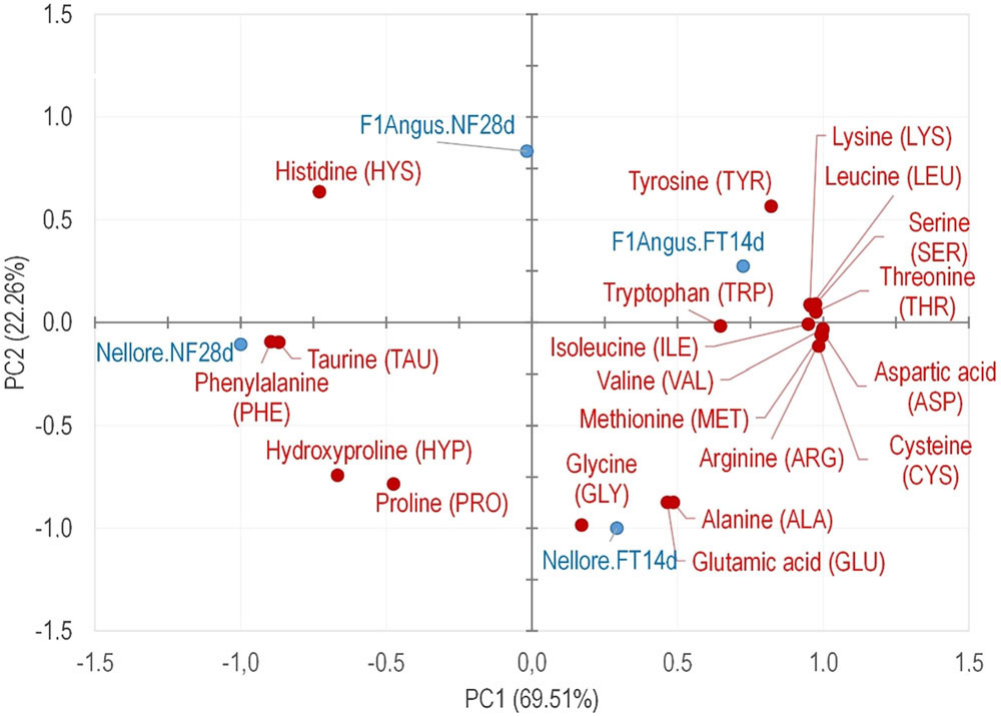
Principal Components Analysis (PCA) of the amino acids (AA) profile of the exudate of dry‐aged grilled beef (*L. lumborum*) from Nellore and crossbreed F1 Angus × Nellore (F1Angus) obtained by accelerated (FT14d) and conventional (NF28d) processes.

The exudate AA profile obtained from dry‐aged grilled meat is shown in Table S3. Of the 20 standard AA, 18 were identified (during analysis, asparagine, and glutamine were hydrolyzed to aspartic acid and glutamic acid, respectively), in addition to the (nonstandard) AAs taurine (TAU) and hydroxyproline (HYP). Interestingly, the HYP, a collagen AA, was identified only in Nellore beef exudates. A PCA graph with the identified AA was generated to discriminate the treatments (Figure 3). The first (PC1) and second (PC2) components represented 91.77% of experimental data variability.

Similarly, the exudate VOC profiles PCA, the AA profile PCA grouped F1Angus in the opposite quadrants than Nellore samples. According to PC1 (largest variability: 69.51%), the Nellore FT14d sample was grouped with F1Angus and Nellore NF28d in an isolated group. This relation is reasonable since the free AA concentration is a substrate for VOC through Maillard and Strecker degradation reactions (Mottram 1998). This result suggested that the exudate played an important role in consumers’ taste perception and preference of meat during cooking. Ribeiro et al. (2024) also reported higher levels of free AAs in exudate samples from dry‐aged meat, reinforcing the direct association between AAs and flavor intensity, as well as their role as precursors for aroma volatiles generated via the Maillard reaction and Strecker degradation. For instance, LEU and ILE can react with dicarbonyl compounds formed during the Maillard reaction to produce key meat odorants such as 2‐ and 3‐methylbutanal.

In the present study, it was possible to achieve a similar AA profile in the exudate using a shorter dry aging time (accelerated process), compared to the conventional process. This result contrasts with previous findings, which emphasize the need for extended aging to promote the accumulation of taste and flavor precursors through protein and lipid degradation (Dashdorj et al. 2016; Bischof et al. 2022). Iida et al. (2016) verified that the free AA content gradually increased during the first 20 days of dry aging, with a more pronounced increase over the following 60 days. Ribeiro et al. (2024) observed higher AA contents in both the meat and the exudate of dry‐aged samples.

### 3.4 Sensory Profile and Acceptance

**FIGURE 4:**
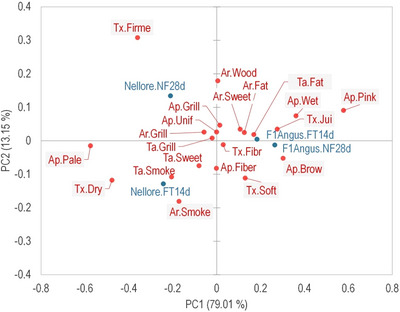
Representation of sensory characteristics from the check‐all‐that‐apply (CATA) applied to dry‐aged grilled beef (*L. lumborum*) from Nellore and crossbreed F1 Angus × Nellore (F1Angus) beef obtained by accelerated (FT14d) and conventional (NF28d) processes. Appearance (Ap.): Pink = pinkish (in the center); Pale = pale; Grill = grilled/barbecue; Unif = uniform; Wet = wet; Fiber = apparent fiber; and Brow = brownish. Aroma (Ar.): Grill = grilled/barbecue; Smoke = smoked; Sweet = sweet/caramel; Fat = fatty/buttery; and Wood = woody. Taste (Ta.): Grill = grilled/barbecue; Smoke = smoked; Sweet = sweet/caramel; and Fat = fatty/buttery. Texture (Tx.): Fibr = fibrous; Soft = soft; Firm = firm; Jui = juicy; and Dry = dry. The sensory characteristics that contributed to discriminating against treatment by the Cochran *Q* test are highlighted in grey.

All attributes of all treatments were well‐accepted by the consumers (Table 2), scoring between 7 (like moderately) and 8 (like very much), except for the appearance of Nellore samples which were scored between 6 (like slightly) and 7. Significant differences (*p* < 0.05) between treatments in the hedonic scores were only observed for appearance, texture, and OA. For the descriptive sensory test, a CA graph was generated using all the characteristics collected from the CATA test, representing 92.16% of the variability in the experimental data (Figure 4). Samples from different aging systems were separated by PC2 (lowest variability, 13.15%), while PC1 discriminated against the genetic group. This is consistent with the acceptance test, where scores did not differ (*p* ≥ 0.05) between meat aged in the conventional or accelerated system from animals of the same genetic group. These results demonstrate the potential of the accelerated process to reduce dry aging time since it does not cause major impacts on the sensory attributes.

**TABLE 2:** Scores (average ± standard deviation) of acceptance test of dry‐aged grilled beef (*L. lumborum*) from Nellore and crossbreed F1 Angus × Nellore (F1Angus) using accelerated (FT14d) and conventional (NF28d) processes.

Breed	Aging	Appearance	Aroma	Taste	Texture	Overall acceptance
F1Angus	FT14d	7.4 ± 1.3^a^	7.2 ± 1.5	7.7 ± 1.1	7.9 ± 1.4^a^	7.8 ± 1.2^a^
	NF28d	7.2 ± 1.5^ab^	7.3 ± 1.3	7.7 ± 1.2	7.9 ± 1.1^a^	7.6 ± 1.1^a^
Nellore	FT14d	6.7 ± 1.6^c^	7.5 ± 1.4	7.7 ± 1.3	7.4 ± 1.3^b^	7.5 ± 1.2^ab^
	NF28d	6.9 ± 1.3^bc^	7.5 ± 1.2	7.4 ± 1.4	7.1 ± 1.6^b^	7.3 ± 1.2^b^

FT14d = frozen (−18°C for 14 days), thawed (4°C for 24 h), and aged samples for 14 days; NF28d = not‐frozen aged samples for 28 days.

^a–c^ Averages followed by distinct letters in the column indicate significant difference (*p* < 0.05) by the Tukey test.

Among 21 characteristics (those with at least 10% of consumers citation) evaluated by the CATA test, 11 did not contribute (*p* ≥ 0.05) to discriminate the samples according to the Cochran *Q* test. Therefore, the consumers perceived a difference in 10 sensory characteristics that contributed to discriminating against treatments (highlighted in gray in Figure 4): four associated with appearance (brownish, pale, wet, and pinkish in the center), one with aroma (smoked) and taste (fatty/buttery), and four related to texture (firm, soft, juicy, and dry). One may notice by PC1 a contrast between the texture characteristics, with “soft” and “juicy” descriptors being positioned on the right, associated with F1Angus samples, and “firm” and “dry” descriptors on the left characterizing Nellore samples. This may explain the higher texture acceptance scores of F1Angus crossbreed meat than Nellore ones. The same trend was observed in appearance characteristics, where descriptors such as “brownish”, “wet”, and “pinkish” in the center were positioned to the right, in opposition to “pale”. Nellore samples appearance was less accepted (*p* < 0.05) than F1Angus samples aged by the accelerated process, although this difference was not significant for conventional‐aged (NF28d) meats. This difference in acceptance may be related to the consumer's perception of a “pale” color that was more associated with the Nellore samples, especially NF28d ones.

Finally, OA scores were higher (*p* < 0.05) for F1Angus than for Nellore meats, regardless of the type of aging system. Nevertheless, the samples Nellore FT14d obtained identical scores (*p* ≥ 0.05) to those of F1Angus (FT14d and NF28d). These results demonstrated the potential for using accelerated aging that, besides reducing the aging time, did not influence the consumers’ acceptance in relation to the evaluated sensory attributes when compared to the conventional method.

### 3.5 Multiple Factor Analysis (MFA)

**FIGURE 5:**
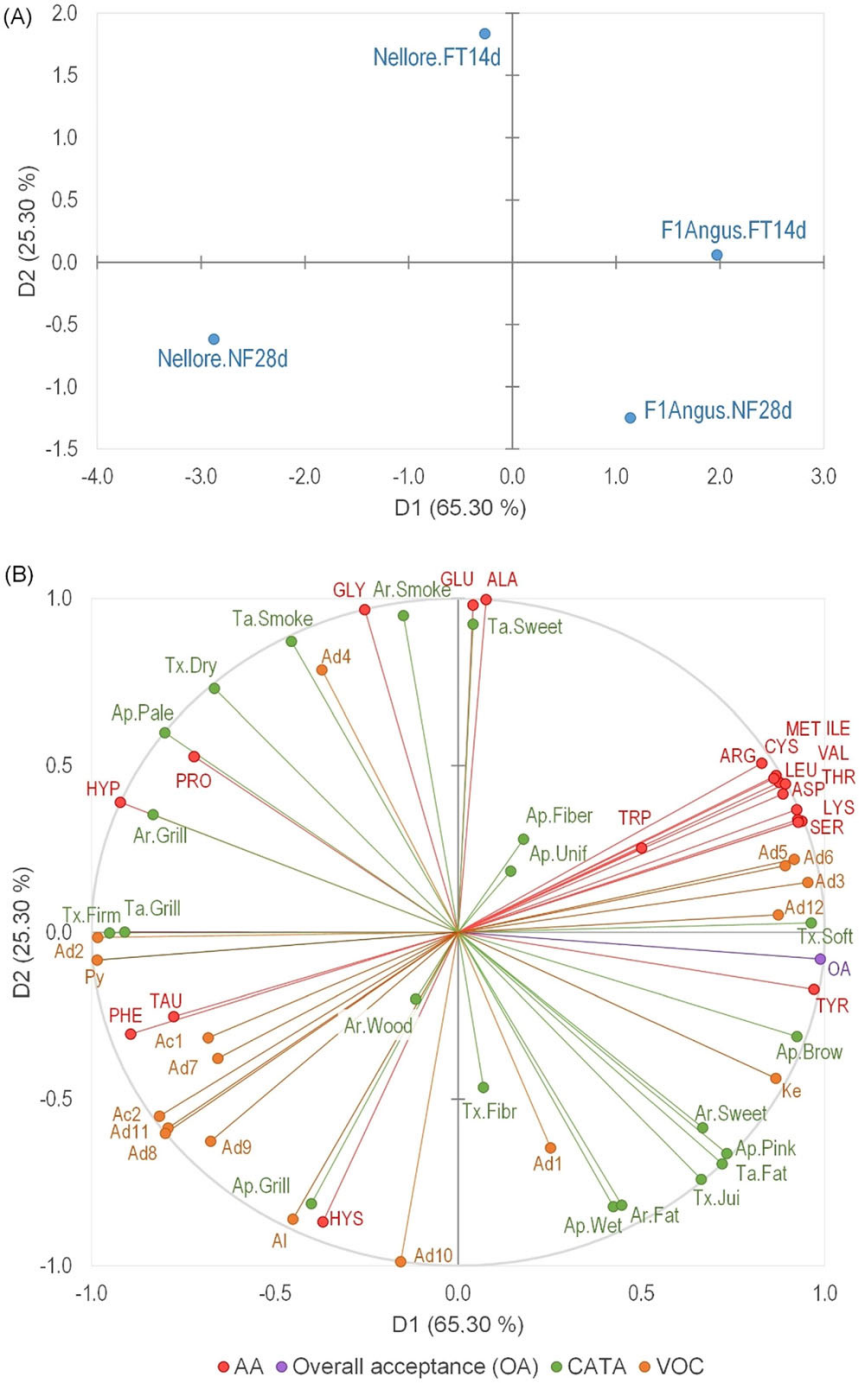
Projections of (A) four treatments (dry‐aged beef of Nellore and crossbreed F1 Angus × Nellore (F1Angus) cattle using the FT14d and NF28d processes) and (B) active variables (VOC, AA, OA, CATA) formed by the dimensions D1 and D2 of the global MFA. Appearance (Ap.): Pink = pinkish; Pale = pale; Grill = grilled/barbecue; Unif = uniform; Wet = wet; Fiber = apparent fiber; and Brow = brownish. Aroma (Ar.): Grill = grilled/barbecue; Smoke = smoked; Sweet = sweet/caramel; Fat = fatty/buttery; and Wood = woody. Taste (Ta.): Grill = grilled/barbecue; Smoke = smoked; Sweet = sweet/caramel; and Fat = fatty/buttery. Texture (Tx.): Fibr = fibrous; Soft = soft; Firm = firm; Jui = juicy; and Dry = dry. Aminoacids (AA): ALA = alanine; ARG = arginine; ASP = aspartic acid; CYS = cysteine; GLU = glutamic acid; GLY = glycine; HYP = hydroxyproline; HYS = histidine; ILE = isoleucine; LEU = leucine; LYS = lysine; MET = methionine; PHE = phenylalanine; PRO = proline; SER = serine; TAU = taurine; THR = threonine; TRP = tryptophan; TYR = tyrosine; and VAL = valine. Volatile organic compounds (VOC): Ac1 = n‐Decanoic acid; Ac2 = n‐Nonanoic acid; Ad1 = Benzaldehyde; Ad2 = Benzaldehyde, 2,3‐dimethyl‐; Ad3 = Benzeneacetaldehyde (Hyacinthin); Ad4 = Butanal; Ad5 = Butanal, 2‐methyl‐; Ad6 = Butanal, 3‐methyl‐; Ad7 = Decanal; Ad8 = Heptanal; Ad9 = Hexanal; Ad10 = Nonanal; Ad11 = Octanal; Ad12 = Propanal, 3‐(methylthio)‐; Al = 1‐Octanol; Ke = 2‐Butanone, 3‐hydroxy (Acetoin); and Py = Pyrazine, 2‐ethyl‐3,5‐dimethyl‐.

The coordinates, respective contributions, and cosine‐squared of variable groups (AA, VOC, descriptive sensory evaluation CATA, and OA) from the two first dimensions of MFA are presented in Table S4. In the global analysis, variables AA, VOC, descriptive sensory evaluation CATA, and OA by the MFA, the first (D1) and second (D2) dimensions represented 90.60% of experimental data total variance (Figure 5). Considering the two dimensions, four groups were formed with each treatment positioned in a different quadrant (Figure 5A). In general, the samples F1Angus (FT14d and NF28d) were evaluated by the consumers by the sensory descriptors of appearance “brownish”, “wet”, and “pinkish in the center”, aroma “sweet/caramel” and “fatty/buttery”, taste “fatty/buttery”, and texture “soft” and “juicy”. The sensory perception of these characteristics seems to be related to the presence of the following AA: cysteine (CYS), tyrosine (TYR), arginine (ARG), ILE, methionine (MET), valine (VAL), LEU, aspartic acid (ASP), threonine (THR), serine (SER), and lysine (LYS). VOC are also related, such as 3‐methylthioproponal (Ad12), 2‐methylbutanal (Ad5), 3‐methylbutanal (Ad6), benzaldehyde (Ad1), Hyacinthine (Ad3), and acetoin (Ke) (Figure 5B). The cited compounds were measured in the grilled beef exudate. Thus, this profile of compounds contributed to the samples’ correlation with overall acceptance (OA).

Among F1Angus beef‐related VOC, the acetoin ketone and the 2‐methylbutanal and 3‐methylbutanal aldehydes had an essential contribution to the taste and aroma of the samples. These compounds correlate to dry‐aged meat's “grilled”, “buttery”, and “nutty” flavors (O'Quinn et al. 2016). Similarly, the relation of those meats with the AA ILE and LEU is important since 2‐methylbutanal and 3‐methylbutanal are products of the Strecker reaction of those compounds (Watanabe et al. 2015). Therefore, the differences in the content of those aldehydes and AA in the exudate of meats from cattle of different genetic groups and aged with different aging systems may play an important role in creating characteristic flavors.

Another characteristic that contributed to the F1Angus aged meat acceptance is its level of marbling fat (9.86 %), which was about 2.3 times the level in Nellore meat (4.23 %). According to Dashdorj et al. (2016), marbling adds taste to the meat because it melts and makes it softer and juicier, generating the characteristic buttery taste. The taste and aroma “fatty/buttery” were attributed to those meat samples. Furthermore, the aged F1Angus beef was more related to UFA, particularly PUFA (Figure 1). According to O'Quinn et al. (2016), besides the increase in the marbling fat level being positively correlated with the flavor, the concentration of MUFA, particularly the oleic acid (C18:1n9), was correlated with the “meaty”, “grilled”, “buttery”, “nutty”, and “taste” intensities. Yet, according to those authors, the increasing concentration of the SFA stearic acid (C16:0) was associated with more intense “bloody/metallic”, “fishy”, “sour”, and “bitter” tastes. In this study, the samples of NF28d Nellore presented a higher relation with SFA and C16:0, but also were the most related with MUFA and C18:1n9. However, those differences in the FA content between treatments were not discrepant in absolute terms.

Nellore samples were more affected by the type of aging system. In the FT14d samples were described by the consumers as having a “smoked” flavor and a “sweet/caramel” and “smoked” taste, along with the AAs GLU, ALA, and GLY, and the VOC butanal (Ad4). As discussed previously, the relation with those AA is interesting because they are associated with the meaty flavor. GLU is the primary AA associated with the umami taste (Iida et al. 2016; Y. H. B. Kim et al. 2016), while the AAs ALA and GLY are associated with the sweet flavor (Y. H. B. Kim et al. 2016). According to Iida et al. (2016), in the meat of high marbling (e.g., the Japanese cattle Wagyu), the umami intensity (induced by the synergic effect of GLU and 5’‐IMP compounds) increased from the 40th day of aging, as it was the shortest dry aging time considered by the authors. It may explain why the AA GLU is not strongly related to the F1Angus samples but reinforces the observation that for the meat of lower marbling, such as Nellore's, accelerated aging enhances their sensory characteristics.

Finally, the conventionally dry‐aged Nellore samples (NF28d) were associated primarily with the descriptor “grilled/barbecue” for appearance, aroma, and taste, and with a “firm” texture. The sensory perception of those characteristics is associated with AAs PHE and TAU, and to VOC hexanal (Ad9), heptanal (Ad8), octanal (Ad11), nonanal (Ad10), benzaldehyde‐2,3‐dimethyl (Ad2), decanal (Ad7), nonanoic acid (Ac2), decanoic acid (Ac1), 1‐octanol (Al), and pyrazine‐2‐ethyl‐3,5‐dimethyl (Py). At higher temperatures, the Maillard reaction products, such as pyrazines, are crucial for developing the “toast” and “nutty” flavors in cooked meats (Watanabe et al. 2015; Ha et al. 2019). Pyrazines are key products of the Maillard reaction, formed through thermal reactions involving AAs, sugars, and sugar degradation products. Compounds such as asparagine, threonine, serine, and glucosamine can contribute to their formation (Watanabe et al. 2015; Ha et al. 2019). Thus, in this study, the consumers might have identified the “toast” taste and aroma as “grilled/barbecue”.

A remarkable feature of Nellore (FT14d and NF28d) samples is its higher association with compounds related to lipid oxidation, such as acids and aldehydes, than F1Angus samples. While the hexanal is formed from the oxidation/degradation of the linoleic FA (C18:2n‐6), heptanal, octanal, and nonanal are derived from the oleic acid (C18:1n‐9) (Van Ba et al. 2012; Domínguez et al. 2019). However, although aldehydes are important taste compounds, they are hardly detected in the aroma and taste. Hence, the importance of each compound in sensory attributes depends on the concentration and the sensibility of perception of each consumer. For instance, the hexanal may promote desirable tastes and, at the same time, produce undesirable tastes in higher concentrations. On the other hand, octanal and nonanal produce desirable tastes (e.g., fatty, sweet, greenish, and oily) essential to cooked meat taste (Van Ba et al. 2012; Ha et al. 2019).

It is important to remark that the sensory evaluation participants were not trained. Therefore, they were unfamiliar with the descriptors commonly used in the literature for dry‐aged meat. Furthermore, dry‐aged meat is still not widely consumed in Brazil, and most participants were unfamiliar with the product's rich taste profile. Nevertheless, the CATA analysis objective described what those consumers perceived and how that perception affected the meat acceptance. Moreover, this study aimed to observe if the differences promoted by the treatments would be noticeable to potential consumers.

Overall, the MFA analysis supports the hypothesis that the accelerated aging method could enhance the sensory profile of meat from low‐marbling cattle (Nellore cattle). Thus, it approximates low‐marbling cattle sensory profile to beef with high marbling fat levels (crossbreed F1Angus). This could be observed in the center of the MFA projections graph (Figure S1), in which the variable groups had distinct influences on the samples. The RV‐coefficient (a multivariate analog of the MFA correlation coefficient) between AA and VOC (0.92) was superior to the RV‐coefficients between AA and CATA (0.56) or between VOC and CATA (0.57). Intermediate RV‐coefficients were observed to the relation of acceptance with AA (0.79), VOC (0.71), and CATA (0.76).

The most significant difference between the MFA and PCA/CA descriptions was found when the samples were described based on the descriptive CATA analysis, particularly for the genetic group. The NF28d Nellore samples are well‐separated from the F1Angus samples (FT14d and NF28d), having the highest sample differentiation in terms of chemical (AA and VOC) and sensory (CATA and OA) profiles. Nevertheless, the FT14d Nellore samples had a chemical profile and OA close to the F1Angus ones.

Y. H. B. Kim et al. (2016) identified 32 metabolites in 21 days dry‐aged beef, including AA and its derivatives (creatine, carnitine, carnosine, and creatinine), known for being highly abundant in meat, sugars (glucose, glucose‐1‐phosphate, glucose‐6‐phosphate), and nucleotides (glutathione, inosine, 5‐monophosphate, guanosine 5‐monophosphate). Although these metabolites enhance the development of taste, either isolated or as chemical reaction precursors, the glutamic acid (GLU), LEU, and ILE levels were higher in dry‐aged beef compared to wet‐aged (Iida et al. 2016; Y. H. B. Kim et al. 2016; Y. H. Kim et al. 2017) and can play an important role in the sensory evaluation of these meats.

The AA LEU and ILE are related to the production of dry‐aged meat odorants 2‐ and 3‐methylbutanal through the Maillard reaction (Koutsidis et al. 2008), while GLU, in synergism with 5‐monophosphate nucleotide (5’‐IMP), is the main component associated with the desirable umami taste (also called meaty taste) (Iida et al. 2016). Moreover, according to Wu et al. (2016), besides GLU, the meaty flavor is associated with β‐alanine (ALA) and glycine (GLY) free AA. In this study, by the PC1, these AA were highly related to Nellore FT14d and F1Angus samples differing from Nellore NF28d. However, by the PC2, the Nellore FT14d sample was more associated with the AA GLY, ALA and GLU, while F1Angus with LEU and ILE. This result agrees with the more intense relation of 2‐methylbutanal and 3‐methylbutanal VOC, characteristic of dry‐aged meat, observed in grilled beef exudates of F1Angus (Figure 2B). Furthermore, the higher relation of Nellore FT14d samples with GLU than Nellore NF28d ones highlights the differences originating from the aging systems.

## 4 Conclusion

The accelerated aging process using freeze/thawing was effective in improving the sensory quality of low‐marbling beef (Nellore), particularly by enhancing the development of maturation‐related flavors without compromising consumer acceptance. This method promoted the formation of desirable volatile compounds, especially aldehydes and pyrazines, contributing to flavor complexity. Despite a moderate increase in lipid oxidation, the levels remained within acceptable limits and did not negatively affect meat quality. In high‐marbling beef (F1 Angus), the process had minimal impact on sensory attributes but preserved a favorable PUFA/SFA ratio and nutritional quality. Moreover, the freeze/thawing approach allowed the achievement of a similar AA profile in the exudate as that obtained through conventional aging, with a distinct VOC profile in both meat and exudate.

## Author Contributions

**Angélica Sousa Guimarães**: conceptualization, methodology, investigation, formal analysis, writing – original draft. **Jéssica Sousa Guimarães**: investigation, formal analysis. **Lorrany Ramos do Carmo**: investigation. **Bruna Fernandes Andrade**: investigation. **Vanelle Maria da Silva**: validation, methodology, writing – review and editing. **Alcinéia de Lemos Souza Ramos**: validation, writing – review and editing. **Eduardo Mendes Ramos**: conceptualization, methodology, supervision, funding acquisition, project administration, writing – review and editing.

## Conflicts of Interest

The authors declared no conflicts of interest.

## Supporting information




**Supplementary Material**: jfds70577‐sup‐0001‐SuppMat.pdf
