# Supplementary material for: Conventional Versus Accelerated Dry‐Aged Meat: Chemical and Sensory Profiles of Longissimus lumborum Muscles From Nellore and Angus × Nellore Crossbreeds
Source: J Food Sci. 2025 Sep 24;90(9):e70577. doi: 10.1111/1750-3841.70577 (PMC12460778; doi:10.1111/1750-3841.70577)
Supplement: Supplementary file 1 — Supplementary Material: jfds70577‐sup‐0001‐SuppMat.pdf [file JFDS-90-0-s001.pdf]

SUPPLEMENTAL INFORMATION

Conventional versus accelerated dry-aged meat: Chemical and sensory profiles of *Longissimus lumborum* muscles from Nellore and Angus×Nellore crossbreeds

Guimarães et al. (2025)

Journal of Food Science

Corresponding author:
Eduardo M Ramos (emramos@ufla.br)
Department of Food Science, School of Agricultural Sciences of Lavras, Federal University of Lavras, Lavras, Minas Gerais, 37200-900, Brazil

Table S1. Fatty acid (FA) profile (% of the normalized area) of Nellore and crossbred F1 Angus x Nellore (F1Angus) dry-aged meat (*L. lumborum*) using accelerated (FT14d) and conventional (NF28d) processes.

		Nellore		F1Angus		
**Fatty acid**		**FT14d**	**NF28d**	**FT14d**	**NF28d**	**SEM**
Myristic	C14:0	/	1.76	0.75	0.95	0.14
Pentadecanoic	C15:0	5.69^{a}	4.65^{b}	4.22^{b}	4.10^{b}	0.34
Palmitic	C16:0	24.32^{b}	27.77^{a}	23.41^{b}	23.28^{b}	0.69
Palmitoleic	C16:1	2.71	2.47	1.81	1.71	0.16
Heptadecanoic	C17:0	3.86^{a}	2.48^{b}	3.47^{a}	3.88^{a}	0.20
Stearic	C18:0	12.43^{b}	14.06^{a}	14.30^{a}	14.38^{a}	0.43
Oleic	C18:1n9c	34.21	34.94	35.16	33.42	0.52
Linoleic	C18:2n6c	10.85^{b}	9.25^{b}	13.05^{a}	14.13^{a}	0.81
Eicosatrienoic	C20:3n6	1.84	/	1.59	2.04	0.12
Aracdonic	C20:4n6	5.01^{a}	3.50^{b}	4.32^{ab}	4.45^{ab}	0.22
Σ Saturated	SFA	46.30^{b}	49.83^{a}	45.78^{b}	46.11^{b}	0.81
Σ Monounsaturated	MUFA	36.92^{a}	37.42^{a}	36.06^{a}	34.28^{b}	0.51
Σ Polyunsaturated	PUFA	16.78^{b}	12.75^{c}	18.16^{ab}	19.61^{a}	1.05
Σ Unsaturated	UFA	53.70^{a}	50.17^{b}	54.22^{a}	53.89^{a}	0.81
Σ Omega-6	n6	16.78^{a}	12.75^{b}	18.16^{a}	19.61^{a}	1.05
	UFA/SFA	1.16^{a}	1.01^{b}	1.19^{a}	1.17^{a}	0.04
	PUFA/SFA	0.36^{b}	0.26^{c}	0.40^{a}	0.43^{a}	0.03

FT14d = frozen (-18 °C for 14 days), thawed (4 °C for 24 h), and aged samples for 14 days; NF28d = not-frozen aged samples for 28 days; SEM = standard error of the mean (n = 12); / = not detected.

$^{(a-c)}$ Means followed by different letters in the row differ ($p < 0.05$).

Table S2. Volatile organic compounds profile (total ions counting $\times 10^4$) of grilled and exudate from dry-aged meat (*L. lumborum*) of Nellore and crossbred F1 Angus x Nellore (F1Angus) using accelerated (FT14d) and conventional (NF28d) processes.

				**Beef**					**Exudate**				
				**Nellore**		**F1Angus**			**Nellore**		**F1Angus**		
**Cod**	**Compound**	**KI_C**	**KI_L**	**FT14d**	**NF28d**	**FT14d**	**NF28d**	**SEM**	**FT14d**	**NF28d**	**FT14d**	**NF28d**	**SEM**
	ACIDS												
Ac1	n-Decanoic acid	1272	1267	/	/	/	/		23.73^{b}	35.61^{a}	/	33.11^{ab}	7.37
Ac2	n-Nonanoic acid	1369	1323	/	/	/	/		28.17^{b}	285.6^{a}	25.25^{b}	135.64^{a}	71.83
	ALCOHOLS												
Al	1-Octanol	1073	1063	7.34^{b}	18.44^{a}	5.91^{b}	15.87^{a}	4.51	/	44.8^{a}	20.58^{b}	29.92^{b}	9.28
	1-Octen-3-ol	978	-	/	19.14	/	7.82	5.40	/	/	/	/	
	1-Pentanol	766	762	/	5.45	/	6.81	2.57	/	/	/	/	
	2-Ethylhexanol	1029	-	/	2.16	/	3.59	2.97	/	/	/	/	
	ALDEHYDES												
Ad1	Benzaldehyde	957	952	23.20	20.57	26.97	31.80	1.83	44.01^{b}	50.52^{b}	37.15^{b}	144.81^{a}	16.93
Ad2	Benzaldehyde, 2,3-dimethyl-	1209	-	/	/	/	/		5.37^{b}	16.48^{a}	/	/	2.67
Ad3	Benzeneacetaldehyde (Hyacinthin)	1040	1036	16.22	/	19.23	/	1.12	304.14^{a}	223.34^{b}	320.27^{a}	322.58^{a}	33.55
Ad4	Butanal	601	-	10.39^{ab}	8.88^{b}	12.07^{a}	11.74^{ab}	0.86	510.94^{a}	405.52^{b}	384.82^{b}	7.10^{c}	58.1
Ad5	Butanal, 2-methyl-	660	654	35.86^{b}	57.74^{a}	33.91^{b}	38.27^{b}	4.70	113.85^{a}	70.34^{b}	112.83^{a}	120.74^{a}	13.3
Ad6	Butanal, 3-methyl-	651	658	26.33^{b}	34.47^{a}	27.97^{b}	31.38^{ab}	2.35	96.6^{a}	59.64^{b}	98.24^{a}	101.30^{a}	11.47
Ad7	Decanal	1200	1201	4.68	5.65	3.48	5.73	0.77	14.91^{c}	34.96^{a}	23.77^{b}	16.40^{bc}	4.05
Ad8	Heptanal	901	901	3.77^{b}	23.65^{a}	7.24^{b}	25.88^{a}	4.66	6.83^{d}	90.25^{a}	16.53^{c}	35.53^{b}	10.5
Ad9	Hexanal	801	801	20.10^{c}	181.03^{a}	25.23^{c}	70.00^{b}	29.23	4.56^{c}	20.75^{a}	10.06^{b}	10.27^{b}	1.89
Ad10	Nonanal	1102	1100	120.60^{b}	282.71^{a}	151.77^{b}	260.49^{a}	34.03	159.2^{c}	546.12^{a}	382.93^{b}	577.22^{a}	92.1
Ad11	Octanal	1001	998	20.60^{b}	65.91^{a}	30.34^{b}	67.60^{a}	10.49	10.67^{c}	205.58^{a}	28.86^{c}	69.52^{b}	26.61
	Pentanal	700	704	/	5.48	2.74	7.67	4.46	/	/	/		
Ad12	Propanal, 3-(methylthio)-	905	901	/	/	/	/		148.53^{b}	/	148.06^{b}	207.25^{a}	17.93
	KETONES												
Ke	2-Butanone, 3-hydroxy (Acetoin)	724	-	9.25^{b}	/	27.23^{a}	23.09^{a}	3.92	/	/	14.51	11.23	2.14
	Octane-2,3-dione	983	-	/	/	/	42.84		/	/	/	/	
	PYRAZINE												

Py	Pyrazine, 2-ethyl-3,5-dimethyl-	1076	-	8.33^{a}	7.78^{a}	2.62^{b}	/	1.25	20.19^{ab}	26.8^{a}	13.57^{c}	18.07^{bc}	3.3
	Σ ACIDS			/	/	/	/		51.90	321.21	25.25	168.75	
	Σ ALCOHOLS			7.34	45.20	5.91	34.10		/	44.80	20.58	29.92	
	Σ ALDEHYDES			281.74	686.09	340.94	550.56		1419.61	1723.50	1563.52	1612.72	
	Σ KETONES			9.25	/	27.23	65.93		/	/	14.51	11.23	
	Σ PYRAZINE			8.33	7.78	2.62	/		20.19	26.80	13.57	18.07	
	Σ TOTAL			306.66	739.07	376.71	650.58		1491.70	2116.31	1637.43	1840.69	

Cod. = abbreviation used to represent each volatile organic compounds; KI_C = Kovats index calculated; KI_L: Kovats index from literature; FT14d = frozen (-18 °C for 14 days), thawed (4 °C for 24 h), and aged samples for 14 days; NF28d = not-frozen aged samples for 28 days; SEM = standard error of the mean (n = 12); / = not detected.

$^{(a\text{-}d)}$ Means followed by different letters in the row, between source (beef or exudate), differ ($p < 0.05$).

Table S3. Amino acids profile (mg/g of protein) of exudates from dry-aged grilled meat (*L. lumborum*) of Nellore and crossbred F1 Angus x Nellore (F1Angus) using accelerated (FT14d) and conventional (NF28d) processes.

		Nellore		F1Angus		
**Amino acid**		**FT14d**	**NF28d**	**FT14d**	**NF28d**	**SEM**
Aspartic acid[1]	ASP	30.19[ab]	24.36[c]	32.30[a]	28.34[b]	1.69
Glutamic acid[1]	GLU	74.21[a]	64.62[bc]	68.82[b]	60.73[c]	2.89
Alanine	ALA	35.22	32.84	33.71	32.39	0.62
Arginine	ARG	21.38	18.01	22.47	20.24	0.95
Cysteine	CYS	6.29	4.24	7.02	5.40	0.60
Phenylalanine	PHE	11.32[b]	59.32[a]	12.64[b]	10.80[b]	1.94
Glycine	GLY	36.48[a]	33.90[b]	33.71[b]	32.39[b]	0.86
Hydroxyproline	HYP	2.52	3.18	/	/	0.33
Histidine	HYS	183.65[b]	199.15[a]	185.39[b]	201.08[a]	4.53
Isoleucine	ILE	12.58	/	12.64	10.80	0.60
Leucine	LEU	21.38	/	23.88	20.24	1.07
Lysine	LYS	35.22	/	39.33	33.74	1.67
Methionine	MET	7.55	5.30	8.43	6.75	0.66
Proline	PRO	22.64[a]	22.25[ab]	21.07[b]	21.59[b]	0.35
Serine	SER	16.35[a]	13.77[b]	16.85[a]	16.19[a]	0.69
Taurine	TAU	6.29	7.42	4.21	6.75	0.69
Tyrosine	TYR	10.06	9.53	11.24	10.80	0.38
Threonine	THR	15.09[a]	12.71[b]	15.45[a]	14.84[a]	0.62
Tryptophan	TRP	2.52[b]	2.12[b]	5.62[a]	1.35[b]	0.94
Valine	VAL	21.38	16.95	22.47	20.24	1.91

FT14d = frozen (-18 °C for 14 days), thawed (4 °C for 24 h), and aged samples for 14 days; NF28d = not-frozen aged samples for 28 days; SEM = standard error of the mean (n = 12); / = not detected.

[(a-c)] Means followed by different letters in the row differ ($p < 0.05$).

[1] Under the analysis conditions, asparagine was hydrolyzed to aspartic acid and glutamine to glutamic acid; therefore, the reported amount of these acids is the sum of those respective components.

Table S4. Coordinates, factors contributions (%), and cosine-squared values (cos^2) of variable groups by the Multiple Factor Analysis (MFA).

	Coordinates		Contributions (%)		cos^2	
**Group of active data**	**D1**	**D2**	**D1**	**D2**	**D1**	**D2**
Amino acid (AA)	0.87	0.43	25.68	33.04	0.68	0.17
Volatile compounds (VOC)	0.86	0.42	25.20	31.58	0.64	0.15
CATA	0.70	0.46	20.36	34.88	0.46	0.20
Overall acceptance (OA)	0.98	0.007	28.76	0.50	0.96	0.00

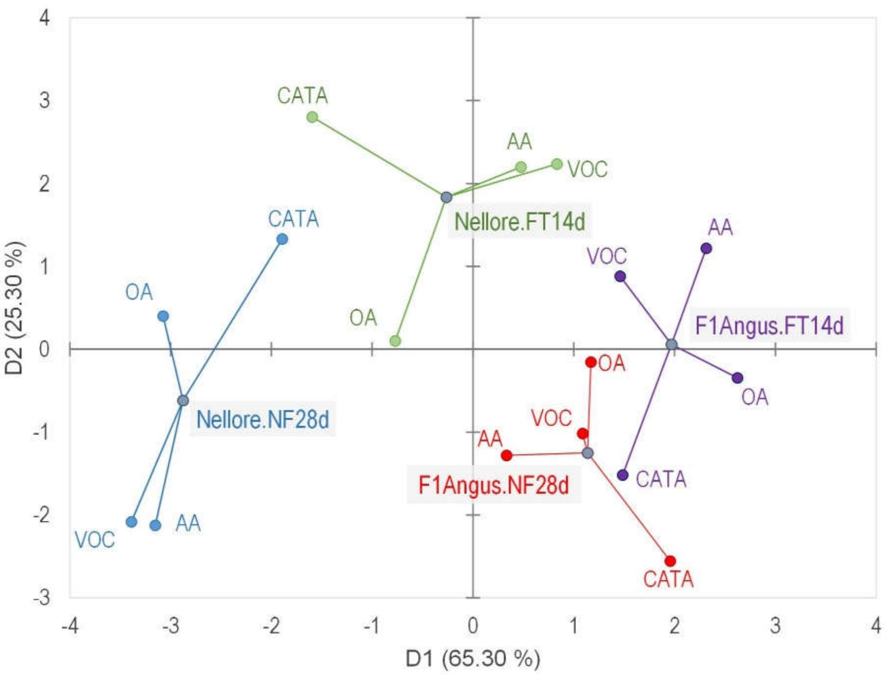

Figure S1. Representation of projected coordinates of variable groups of global MFA to dry-aged grilled beef (*L. lumborum*) from Nellore and crossbreed F1 Angus×Nellore (F1Angus) obtained by accelerated (FT14d) and conventional (NF28d) processes. VOC = volatile organic compounds; AA = amino acids; OA = overall acceptance; CATA = check-all-that-apply.
